# Diagnostic Efficacy of Olfactory Function Test Using Functional Near-Infrared Spectroscopy with Machine Learning in Healthy Adults: A Prospective Diagnostic-Accuracy (Feasibility/Validation) Study in Healthy Adults with Algorithm Development

**DOI:** 10.3390/diagnostics15192433

**Published:** 2025-09-24

**Authors:** Minhyuk Lim, Seonghyun Kim, Dong Keon Yon, Jaewon Kim

**Affiliations:** 1 Institute for Artificial Intelligence, N.CER Co., Ltd., Gwangju 61005, Republic of Korea; 2 Center for Digital Health, Medical Science Research Institute, Kyung Hee University Medical Center, Kyung Hee University College of Medicine, Seoul 02447, Republic of Korea

**Keywords:** YSK olfactory function, functional near-infrared spectroscopy, machine learning

## Abstract

**Background/Objectives**: The YSK olfactory function (YOF) test is a culturally adapted psychophysical tool that assesses threshold, discrimination, and identification. This study evaluated whether functional near-infrared spectroscopy (fNIRS) synchronized with routine YOF testing, combined with machine learning, can predict YOF subdomain performance in healthy adults, providing an objective neural correlate to complement behavioral testing. **Methods**: In this prospective diagnostic-accuracy (feasibility/validation) study in healthy adults with algorithm development, 100 healthy adults completed the YOF test while undergoing prefrontal/orbitofrontal fNIRS during odor blocks. Feature sets from ΔHbO/ΔHbR included time-domain descriptors, complexity (Lempel–Ziv), and information-theoretic measures (mutual information); the identification task used a hybrid attention–CNN. Separate models were developed for threshold (binary classification), discrimination (binary classification), and identification (binary classification). Performance was summarized with accuracy, area under the curve (AUC), F1-score, and (where applicable) sensitivity/specificity, using participant-level cross-validation. **Results**: The threshold classifier achieved accuracy 0.86, AUC 0.86, and F1 0.86, indicating strong discrimination of correct vs. incorrect threshold responses. The discrimination model yielded accuracy 0.75, AUC 0.76, and F1 0.75. The identification model (attention–convolutional neural network [CNN]) achieved accuracy 0.88, sensitivity 0.86, specificity 0.91, and F1 0.88. Feature-attribution (e.g., SHapley Additive exPlanations [SHAP]) provided interpretable links between fNIRS features and task performance for threshold and discrimination. **Conclusions**: Olfactory-evoked fNIRS signals can accurately predict YOF subdomain performance in healthy adults, supporting the feasibility of non-invasive, portable, near–real-time olfactory monitoring. These findings are preliminary and not generalizable to clinical populations; external validation in diverse cohorts is warranted. The approach clarifies the scientific essence of the method by (i) aligning psychophysical outcomes with objective hemodynamic signatures and (ii) introducing a feature-rich modeling pipeline (ΔHbO/ΔHbR + Lempel–Ziv complexity/mutual information; attention–CNN) that advances prior work.

## 1. Introduction

The olfactory sense, one of the five human senses, plays a critical role beyond the perception of scents. It functions as an early warning system for hazardous substances, influences appetites and satiety, and contributes to emotional memory and social communication [1]. Despite these essential functions, olfactory dysfunction (OD) is both prevalent and frequently under-recognized in clinical practice [2]. A previous study estimated that approximately 20–25% of adults experience some degree of OD, with prevalence increasing markedly with age [2]. Importantly, growing evidence indicates that OD is not only a symptom of upper respiratory tract infections but also an early clinical marker of neurodegenerative diseases, including Alzheimer’s and Parkinson’s disease, a predictor of cognitive decline [1,3,4], and even a risk factor for increased mortality in older adults [5].

Objective assessment of olfactory function is commonly performed using psychophysical tests such as the University of Pennsylvania Smell Identification Test (UPSIT; Sensonics International, Haddon Heights, NJ, USA)., Sniffin’ Sticks test, and more recently, the culturally adapted Olfactory function was assessed using the YSK Olfactory Function Test (YOF; Korean Version; RHICO Medical Co., Seoul, Republic of Korea) [6,7,8]. The YOF test, specifically developed for Korean populations, incorporates odorants that are familiar, safe, and culturally relevant [7]. These instruments evaluate three primary domains of olfactory function: threshold (ability to detect), discrimination (the ability to distinguish between different odors), and identification (the ability to recognize specific odors). While these tests are well-validated, they are inherently behavioral and therefore depend heavily on participants’ attention, motivation, and cognitive capacity. Consequently, they rely largely on subjective responses, which can make it difficult to determine whether an examinee truly perceives an odor or is reporting symptoms in the absence of perception. This problem is especially salient in heterogeneous patient groups (e.g., individuals with post–COVID-19 sequelae), who often face practical challenges during testing. To date, there has been limited research integrating psychophysical olfactory tests with objective neurophysiological markers to mitigate this subjectivity, and standardized pathways to align reported symptoms with test performance remain underdeveloped.

Neuroimaging methods offer a complementary avenue for assessing olfaction by directly measuring brain activity associated with odor processing. Functional near-infrared spectroscopy (fNIRS) is an emerging neuroimaging modality that non-invasively measures cortical hemodynamic responses via changes in oxygenated and deoxygenated hemoglobin concentrations [9]. fNIRS offers several practical advantages, including portability, affordability, and suitability for repeated or ambulatory measurements. Previous studies have shown that olfactory stimulation elicits significant activation in the orbitofrontal cortex, dorsolateral prefrontal cortex, and other prefrontal regions, which are critical for olfactory perception and higher-order executive processing [9,10,11].

While fNIRS has been used to detect cognitive decline in patients with Alzheimer’s disease, mild cognitive impairment, and autism spectrum disorders, [12,13,14] few studies have attempted to evaluate whether fNIRS can predict detailed psychophysical olfactory function scores in healthy adults. A recent post hoc analysis by Kim et al. (2023) demonstrated that olfactory-stimulated functional magnetic resonance imaging (fMRI), combined with machine learning, can identify mild cognitive impairment and early Alzheimer’s disease [15]. Extending this concept, integrating fNIRS data with machine learning provides an opportunity to decode nuanced patterns of neural activity and potentially approximate psychophysical test performance. Machine learning models can capture complex, non-linear relationships between cortical hemodynamics and olfactory outcomes, offering a path toward portable, non-invasive olfactory diagnostics [12,13].

To address this gap, the present study aimed to explore whether fNIRS signals collected during olfactory stimulation could accurately predict YOF test outcomes in healthy adults. By applying machine learning algorithms to hemodynamic responses, we evaluated the feasibility of non-invasively estimating threshold, discrimination, and identification scores, contributing to the development of wearable olfactory diagnostics.

Building on our prior work and related literature [6], olfactory stimulation elicits measurable hemodynamic responses in prefrontal/orbitofrontal regions that can distinguish healthy individuals from patients with neurodegenerative conditions (e.g., Alzheimer’s disease). Leveraging this principle, we synchronized fNIRS acquisition with routine YOF testing in order to quantify how cortical signals relate to, and potentially predict, psychophysical outcomes using machine learning models.

We prespecified the following hypotheses: (1) threshold—prefrontal fNIRS features will classify threshold task performance above chance (target area under the curve [AUC] ≥ 0.70); (2) discrimination—models incorporating complexity/information-theoretic features (e.g., Lempel–Ziv complexity, mutual information) will classify discrimination performance with balanced accuracy (target AUC ≥ 0.70); and (3) identification—an attention-based model will classify identification accuracy at a high level (target AUC ≥ 0.80).

We prospectively synchronized fNIRS acquisition with each YOF subtask and trained models to predict YOF outcomes from the concurrent prefrontal/orbitofrontal signals, positioning fNIRS + machine learning as an augmentation to behavioral testing in healthy adults.

## 2. Materials and Methods

### 2.1. Study Design

We conducted a prospective diagnostic-accuracy (feasibility/validation) study in healthy adults with algorithm development, evaluating whether time-locked prefrontal/orbitofrontal fNIRS signals acquired during YOF testing can predict psychophysical outcomes for threshold, discrimination, and identification.

### 2.2. Participants

A prospective diagnostic accuracy study using algorithm development was conducted in a laboratory setting. Participants were recruited between 25 November and 18 December 2024. A total of 100 healthy adult participants (age range 21–76 years; mean ± standard deviation (SD), 50.9 ± 11.4) were enrolled. Study flow is shown in Supplementary Figure S1. All participants reported no history of neurological, psychiatric, or olfactory disorders and completed questionnaires, the YOF test, and concurrent fNIRS measurements. Inclusion criteria included volunteers aged 20–90 years who were free of systemic diseases and voluntarily agreed to participate. Exclusion criteria included: being pregnant or lactating or planning to become pregnant within the next 6 months; participating in the same study within the past month; using medications that could affect brain function; currently receiving psychiatric treatment or using psychiatric medications; using cosmetics or medications with similar efficacy applied in the study area within the month prior to the start of the study; having chronic diseases (e.g., asthma, diabetes, hypertension); working at a clinical research institution; and any other circumstances deemed unsuitable by the researcher. No additional demographic variables were collected. Written informed consent was obtained in accordance with the Declaration of Helsinki, and this protocol was approved by the Institutional Review Board of the Korea Biomedical Research Institute (protocol code: F-2024-024-01; approval date: 24 October 2024).

### 2.3. YOF Test

Each participant underwent the YOF test, a validated olfactory assessment tool culturally adapted for the Korean population [6,7]. The test evaluates three subdomains of olfactory function: (1) threshold: detection of phenyl-ethyl alcohol at varying dilutions; (2) discrimination: differentiation of similar-smelling odor pairs; and (3) identification: recognition of 12 odors, including culturally familiar scents.

A total TDI (threshold + discrimination + identification) score was calculated by summing the three subdomain scores (range: 1–36). Participants were classified as normosmic if their TDI score exceeded 21, hyposmic if the score ranged from 14.5 to 21, and anosmic if the score was 14.5 or below, based on established YOF criteria [6,7].

### 2.4. Identification Subgroup Analysis Based on Odorant Molecular Structure

To explore the relationship between olfactory identification and the chemical properties of odorants, the 12 odorants used in the YOF identification subtest were further classified into two subgroups based on similarity in molecular structure and functional group composition. Group A included simpler aromatic compounds (e.g., peach, chocolate, cinnamon, baby powder), while Group B comprised odorants with more complex or pungent molecular structures (e.g., herbal medicine, spearmint, naphthalene, grilled meat), as informed by prior studies [6].

### 2.5. fNIRS Measurement

Prefrontal fNIRS signals were recorded during YOF odor blocks using a dual-wavelength, continuous-wave system covering the orbitofrontal and dorsolateral prefrontal cortices. Odor presentation was time-locked to the acquisition via event markers to permit stimulus-locked epoching. Device layout, optode montage, sampling parameters, stimulus timing, and preprocessing steps are described in detail in 2.5–2.6, and feature extraction procedures in 2.7.

### 2.6. fNIRS Acquisition

fNIRS data were recorded using a commercially available N.CER system (model N1; N.CER Co., Ltd., Seoul, Republic of Korea), with optodes positioned over the prefrontal cortex—covering the dorsolateral prefrontal and orbitofrontal regions—according to the International 10–20 System [16]. Dual-wavelength acquisition at 730 and 850 nm was used, sampled at 10 Hz, with source–detector separations of 4.0, 3.5, and 3.0 cm. To minimize superficial contamination, emitters and detectors were placed approximately 1 cm superior to the supraorbital ridge. This configuration enabled continuous monitoring of cortical hemoglobin oxygenation. In this study, the FP1 and FP2 positions were localized above the eyebrows in accordance with the International 10–20 System used for electroencephalography [16]. Odorants from the YOF test were administered using a block design consistent with the clinical test; event markers were logged at odor onset on the acquisition console to synchronize stimulus timing with the fNIRS signal. Raw optical density signals were converted to changes in oxygenated and deoxygenated hemoglobin using the modified Beer–Lambert law [17]. Preprocessing included motion-artifact correction, baseline normalization, and band-pass filtering (0.01–0.20 Hz) [9,10].

### 2.7. Preprocessing

Raw fNIRS time series were preprocessed to mitigate physiological and motion artifacts. The pipeline comprised (1) visual and automated screening to exclude bad channels according to predefined quality criteria (e.g., excessive spikes, low signal-to-noise ratio, or missing segments); (2) band-pass filtering (0.01–0.20 Hz) to attenuate baseline drift and high-frequency noise; (3) wavelet- or spline-based correction for motion artifacts; and (4) epoching time-locked to stimulus onsets. After preprocessing, hemoglobin-concentration time series (ΔHbO, ΔHbR, and HbT) were baseline-corrected to the pre-stimulus interval on a channel-wise basis.

### 2.8. Feature Extraction

A comprehensive set of features was computed on each stimulus epoch for each channel and hemoglobin species. Feature categories included: Time-domain descriptors: number of peaks, curve length, peak amplitude and latency, and other waveform morphology measures; complexity measures: Lempel–Ziv complexity (LZC) to quantify local signal complexity; nonlinear/information measures: mutual information between channels to capture inter-channel dependencies; time–frequency/wavelet features: wavelet-based statistics computed from discrete wavelet decompositions to capture transient spectral properties; and additional summary statistics (means, variances, skewness, kurtosis) computed for each epoch. Features were computed per channel and then aggregated or indexed by channel/hemoglobin mapping. Prior to model training, features were standardized (z-scored) based on the training set distribution.

### 2.9. Machine Learning Analysis

- Threshold and discrimination used established classifiers (logistic regression, random forest, multilayer perceptron), whereas identification employed a lightweight attention–CNN adapted for multichannel fNIRS time series. For each stimulus epoch, multichannel feature vectors comprised time-domain descriptors, LZC, information-theoretic measures (mutual information), and wavelet-based statistics computed from ΔHbO/ΔHbR validation, class imbalance handling, and evaluation.
  To address class imbalance, repeated trials within participants, and the modest sample size, we used participant-level cross-validation rather than fixed train/validation/test splits. For all three subdomains, model training and evaluation used 10-fold GroupKFold (group = participant) repeated five times with different random seeds. Within each training fold, we applied class-balanced resampling: for threshold and discrimination, the majority class was randomly under-sampled to match the minority class; and for identification, class weights were used in the cross-entropy loss. Hyperparameters were tuned by an inner 5-fold CV restricted to the training fold only. No participant contributed trials to more than one outer fold.
- Uncertainty quantification and reporting.
  We estimated uncertainty using a 2000-sample stratified, participant-grouped bootstrap to obtain 95% confidence intervals (CIs) for all metrics. Results are reported as mean ± SD across outer folds together with bootstrap 95% CIs (and, where applicable, a participant-level hold-out set is reported separately).
- Models, metrics, and interpretability.
  We evaluated random forest, logistic regression, and multilayer perceptron baselines; for identification we additionally assessed a hybrid attention–convolutional neural network (CNN). Classification metrics included accuracy, AUC, F1-score, sensitivity, and specificity. Model interpretability used SHapley Additive exPlanations (SHAP) for tree-based models and attention-weight visualization for the deep model.
- Software and reproducibility.
  All analyses were performed in Python, version 3.11 (Python Software Foundation, Wilmington, DE, USA), with scikit-learn, version 1.7.2 (scikit-learn developers, open-source project, France); XGBoost, version 3.0.5 (XGBoost community, open-source project, USA); Keras, version 3.11.3 (Keras developers, open-source project, USA); SHAP, version 0.48.0 (SHAP community, open-source project, USA); and imbalanced-learn, version 0.14.0 (imbalanced-learn developers, open-source project, France).

Model training and evaluation used 10-fold GroupKFold (by participant) repeated five times; uncertainty was quantified with a 2000-sample stratified, participant-grouped bootstrap to obtain 95% confidence intervals. Class imbalance was handled within training folds, and no participant contributed trials to more than one fold

### 2.10. Identification Task

For the olfactory identification task, we implemented an attention-augmented convolutional neural network (attention-CNN) that directly used preprocessed multi-channel fNIRS epochs as input: multichannel epoch tensors; architecture: a sequence of 1D convolutional layers to extract local temporal features, followed by attention modules to weight informative temporal segments and channels, and fully connected layers for classification; dropout and batch normalization were used to regularize training; training: and the network was trained using categorical cross-entropy with an adaptive optimizer. Early stopping on validation loss and learning-rate reduction on plateau were used to avoid overfitting. Class weights were applied when appropriate.

### 2.11. Sample Size and Power

Sample size and power. We planned a target sample size of approximately 100 healthy adults for algorithm development and internal validation. This pragmatic choice was informed by prior patient-level diagnostic trials of olfactory-stimulated fNIRS, which enrolled *n* = 97 and *n* = 168 participants, respectively, and achieved strong discrimination against cognitive-impairment phenotypes (e.g., AUC ≈ 0.85–0.91 depending on the endpoint and comparator).

In the absence of directly comparable effect estimates for healthy adults on psychophysical olfactory outcomes, we adopted a conservative planning target of AUC = 0.70–0.75 (vs. 0.50 under the null), α = 0.05, and ≥80% power. By analogy with two-group calculations used in related olfactory fNIRS trials—where ~15–17 participants per class were estimated to yield ~75–90% power to detect large between-group oxygenation differences at conventional significance levels—an overall sample near *n* ≈ 100 (with balanced splits across classification tasks) was deemed adequate for our primary model-based analyses. To quantify uncertainty, we report cross-validated performance with bootstrap 95% CIs and, where applicable, hold-out test estimates; this approach yields precision that is consistent with the above planning assumptions.

### 2.12. Blinding and Leakage Control

Data leakage prevention. To minimize operational and analytic leakage, role- and time-based separation of information was enforced. Examiners administering the YOF procedures had no access to the fNIRS acquisition console or raw signals, and composite YOF scores were graded by an independent rater, such that examiners remained unaware of test results. Participants underwent the assessment only; no feedback about performance or results was provided during or after the session. The analysis plan—including preprocessing, feature definitions, model families/hyperparameters, cross-validation, and primary metrics—was finalized and time-stamped prior to label access. Item-level correctness and composite scores, graded offline by the independent rater, and the raw fNIRS files, stored on the device main unit and retrieved after data collection, were subsequently linked by a data manager using subject identifiers under a written SOP. Analysts were then provided with a de-identified analysis dataset after plan lock; the hold-out test set remained sequestered until model selection and tuning were completed. Additionally, all cross-validation used GroupKFold at the participant level, ensuring that trials from any given individual appeared in only one fold and never in both training and validation/test partitions. Reporting adheres to STARD 2015 and relevant TRIPOD-AI items; completed checklists are provided in Supplementary Checklists S1 and S2.

## 3. Results

### 3.1. Threshold Classification Performance

**Figure 1:**
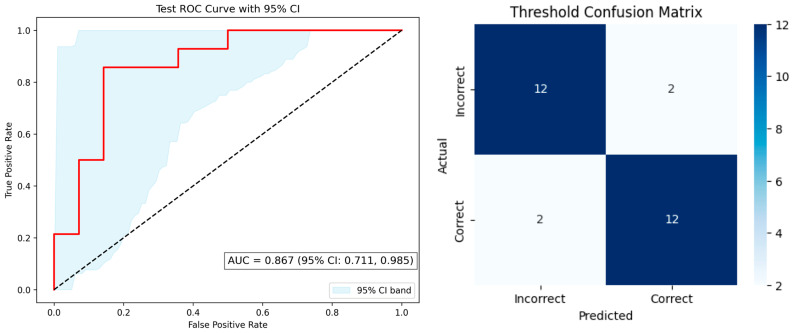
**Left**: Receiver operating characteristic (ROC) curve for threshold prediction. The red line indicates the ROC curve, and the dashed diagonal line represents the reference line for random classification (AUC = 0.867 [95% CI, 0.711–0.985]). The shaded area denotes the 95% confidence interval. **Right**: Confusion matrix for threshold classification (12 true negatives, 12 true positives, 2 false positives, 2 false negatives).

A total of 100 healthy adult participants (age range 21–76 years; mean ± SD, 50.9 ± 11.4) were enrolled (Table 1 and Figure S1). The XGBoost classifier for threshold-level olfactory response prediction achieved an accuracy of 0.86, with both sensitivity and specificity at 0.86 ± 0.04 (95% CI 0.78–0.92), and an AUC of 0.86 ± 0.04 (95% CI 0.78–0.92) (Figure 1, left), indicating strong discriminatory power. The confusion matrix (Figure 1, right) demonstrated balanced results: 12 true positives, 12 true negatives, and 2 false predictions per class.

**Figure 2:**
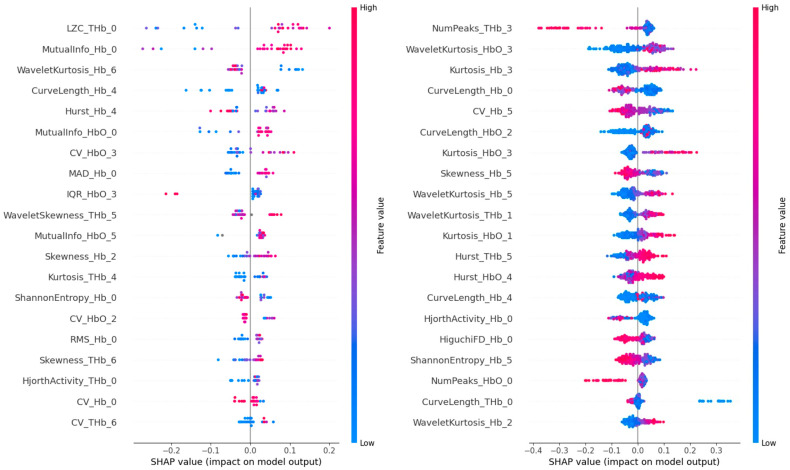
**Left**: SHAP summary plot of the XGBoost threshold model. **Right**: SHAP summary plot of the XGBoost discrimination model.

SHAP analysis (Figure 2, left) identified NumPeaks_THb_3, WaveletKurtosis_HbO_3, and CurveLength_Hb_0 as the top contributors. These features, mainly derived from orbitofrontal and dorsolateral prefrontal regions, suggest that signal peak frequency and waveform complexity play a critical role in threshold-level odor detection (Table S1).

### 3.2. Discrimination Classification Performance

**Figure 3:**
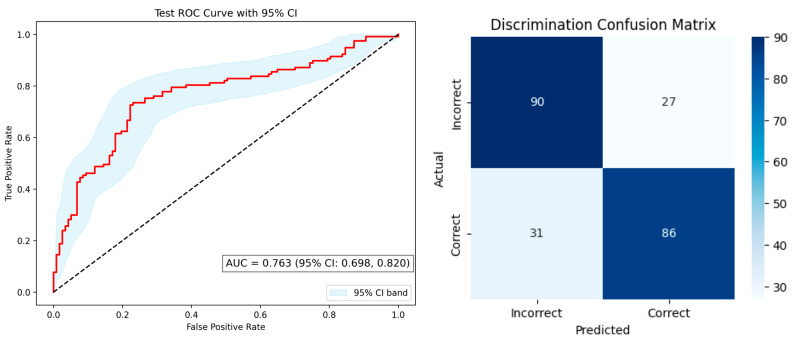
**Left**: Receiver operating characteristic (ROC) curve for discrimination prediction. The red line shows the ROC curve, and the dashed diagonal line represents random classification (AUC = 0.763 [95% CI, 0.698–0.820]). The shaded area indicates the 95% confidence interval. **Right**: Confusion matrix for discrimination classification.

The discrimination task model, also using XGBoost, achieved an accuracy of 0.75, sensitivity of 0.74, and specificity of 0.77, and an AUC = 0.763 ± 0.065 (95% CI: 0.698–0.820) (Figure 3, left). The confusion matrix (Figure 3, right) showed 86 true positives, 90 true negatives, 27 false positives, and 31 false negatives, reflecting moderate classification performance.

SHAP analysis (Figure 2, right) indicated that LZC_THb_0, MutualInfo_Hb_0, and WaveletKurtosis_Hb_6 were the most influential features. These metrics reflect local signal complexity and non-linear relationships in hemoglobin dynamics, highlighting their importance in differentiating similar odor stimuli.

### 3.3. Identification Classification Performance

**Figure 4:**
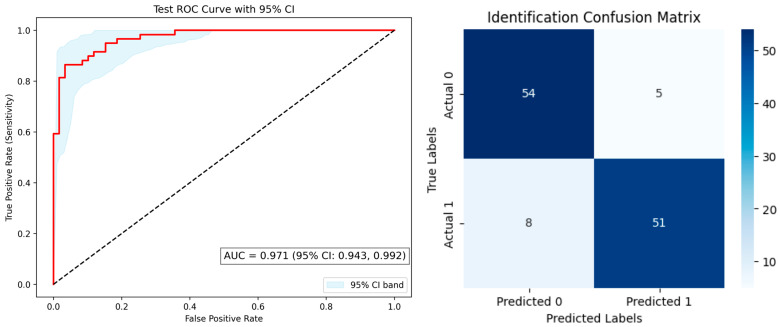
**Left**: Receiver operating characteristic (ROC) curve (red) with 95% confidence interval (CI) band. The area under the curve (AUC) was 0.971 ± 0.028 (95% CI, 0.943–0.992). The dashed diagonal line indicates the reference for random classification. **Right**: Confusion matrix for identification classification.

For olfactory identification, the attention-based CNN model exhibited the highest classification performance, with an AUC = 0.971 ± 0.028 (95% CI: 0.943–0.992), sensitivity of 0.86, specificity of 0.91, and F1-score of 0.88 (Figure 4). Unlike the threshold and discrimination models, SHAP analysis was not applied to the identification model due to its deep learning architecture and interpretability limitations in CNN-attention frameworks.

## 4. Discussion

In this prospective diagnostic-accuracy (feasibility/validation) study in healthy adults, we investigated whether fNIRS signals collected during olfactory stimulation, combined with machine learning, could predict psychophysical olfactory function as measured by the YOF test in healthy adults. Specifically, we evaluated the classification performance of machine learning models trained on hemodynamic responses from the prefrontal cortex to predict subdomain outcomes of the YOF test—threshold, discrimination, and identification. Our findings revealed that fNIRS-based models achieved moderate-to-high predictive performance across all subdomains, with the strongest results observed in threshold and identification.

The threshold model, implemented via an XGBoost classifier, achieved an accuracy of 0.86, AUC of 0.8622, and F1-score of 0.86, Importantly, SHAP analysis revealed that peak-related and kurtosis-based time-series features from orbitofrontal and dorsolateral prefrontal channels contributed significantly to this predictive performance, corroborating prior neuroimaging studies emphasizing the orbitofrontal cortex’s role in early olfactory processing [18].

The discrimination model yielded an accuracy of 0.75, AUC of 0.762, and F1-score of 0.75, with balanced sensitivity and specificity. The confusion matrix confirmed relatively even distribution of correct and incorrect predictions. SHAP analysis identified that LZC and information-theoretic (mutual information metrics) were key predictive features, suggesting that neural signal complexity and temporal variability are critical for discrimination. These findings suggest that odor discrimination tasks engage prefrontal mechanisms related to temporal complexity and signal diversity.

The most notable performance was observed for the identification model, which employed an Attention-CNN hybrid architecture. This model achieved the highest predictive performance, with an accuracy of 0.88, sensitivity of 0.86, specificity of 0.91, and F1-score of 0.88. While feature attribution techniques such as SHAP were applied to the threshold and discrimination models, they were not used for the identification model due to the inherent complexity of deep learning interpretability. Nonetheless, these findings extend prior research using fNIRS to detect olfactory-related brain activity in populations with cognitive decline [12].

Taken together, these findings indicate that fNIRS signals carry sufficient information to approximate psychophysical olfactory function even within a healthy, normosmic population. This is particularly noteworthy because prior studies using fNIRS and other neuroimaging modalities have largely focused on binary classification of disease states, such as differentiating Alzheimer’s patients from controls, rather than estimating detailed subdomain performance in healthy individuals [12]. The present work therefore extends the potential application of fNIRS from disease detection to nuanced sensory profiling, opening avenues for real-time, non-invasive olfactory monitoring.

### 4.1. Possible Mechanism

Our findings are consistent with existing knowledge of olfactory neuroanatomy and functional neuroimaging. While primary olfactory cortex receives direct input from the olfactory bulb, higher-order processing occurs predominantly in the dorsolateral prefrontal cortex [19]. According to recent reviews of fNIRS studies on olfaction, the dorsolateral prefrontal cortex is frequently observed to be activated alongside the orbitofrontal cortex and the inferior frontal gyrus during both olfactory stimulation and imagery [11]. In our study, the contribution of kurtosis features from dorsolateral prefrontal cortex channels to the threshold model suggests that the morphological characteristics of neural responses to the intensity and temporal structure of olfactory stimuli are important for sensitivity discrimination.

Furthermore, the prominence of complexity-based metrics, including LZC and mutual information as significant predictors in the discrimination model may indicate that discrimination tasks demand greater complexity and variability in neural signals compared with simple detection [20,21]. In various neuroimaging studies, complexity measures such as LZC and entropy have been used to characterize differences between healthy individuals and those with cognitive impairments [20,22]. It further underscores the non-linear dynamics of prefrontal activity during odor discrimination. Prior fMRI and EEG studies have similarly demonstrated that prefrontal regions exhibit increased variability and connectivity when individuals engage in sensory discrimination or recognition tasks, supporting our interpretation that these metrics reflect higher cognitive load and integrative processing [23]. In the identification task, high predictive accuracy using an Attention-CNN architecture suggests that integrated activity across multiple prefrontal regions encodes the perceptual and cognitive processes necessary for odor recognition. These results highlight that fNIRS signals capture both low-level sensory detection and higher-order cognitive processing of olfactory stimuli.

### 4.2. Implications

The results of this study have several important implications. These results highlight the potential of fNIRS-based decoding of olfactory performance in digital health applications [24]. NIRS-based decoding of olfactory performance offers a non-invasive, objective, and potentially portable alternative to traditional behavioral tests, which can be influenced by participant attention, motivation, or cognitive function. This is particularly relevant for aging populations and clinical groups, where conventional tests may underestimate olfactory function due to non-sensory factors [25]. Early detection olfactory decline could facilitate timely interventions. Second, fNIRS-based olfactory monitoring has the potential to serve as a digital biomarker for early detection of neurodegenerative disorders, enabling longitudinal tracking of cortical function before overt clinical symptoms manifest. Third, integrating machine learning with fNIRS expands the interpretive capacity of neuroimaging, allowing the extraction of subtle spatiotemporal patterns that reflect individual differences in sensory perception. This approach could be generalized to other sensory domains, supporting a broader agenda of non-invasive, neural-based functional diagnostics [26].

### 4.3. Limitations

Several limitations of the study warrant discussion. First, the cohort consisted solely of healthy, normosmic adults aged 21–76 years and showed a potential sex imbalance (Table 1), limiting generalizability and possibly underestimating the model’s discriminative capacity in olfactory-impaired populations. Exploratory sex-stratified summaries are provided in Supplementary Table S1. Second, the use of binary classification for discrimination and identification tasks may not capture, thus underestimate the continuous spectrum of real-world olfactory performance. Third, fNIRS is inherently sensitive to motion artifacts and extracerebral blood flow, which may introduce noise into signal interpretation [27]. Fourth, while our models achieved high accuracy, deep learning architectures such as the Attention-CNN used for identification lack full interpretability, limiting insight into the precise neural mechanisms underlying predictive success. Finally, the cultural specificity of the YOF test limits generalizability to non-Korean populations. As a mitigation, subgroup performance by sex was prespecified as an exploratory sensitivity analysis, and all findings should be interpreted as preliminary pending external validation in demographically diverse cohorts. Because the cohort consisted of healthy, normosmic adults tested in a laboratory setting, results are not generalizable to clinical populations (e.g., patients with hyposmia/anosmia or neurodegenerative disease) or other real-world settings. External validation in independent, demographically diverse cohorts and clinical environments is warranted. The proposed fNIRS + machine-learning approach offers portability, non-invasiveness, and objective neural readouts, enabling near–real-time assessment. Compared with purely psychophysical tests (e.g., UPSIT, Sniffin’ Sticks, YOF test), it reduces dependence on attention, motivation, and cognitive status and provides a physiological correlate of performance. Relative to fMRI, it requires lower infrastructure and cost and is more feasible for bedside or ambulatory use; compared with EEG, it affords direct hemodynamic measurement over prefrontal regions, albeit with lower spatial resolution than fMRI and limited depth sensitivity. To minimize publication bias and analytical flexibility, the study prespecified primary metrics and analysis procedures, implemented an analysis-plan lock prior to label access, and reports all subdomain models (including less favorable results) according to the STARD 2015/TRIPOD-AI items. Software versions and parameter settings are provided in the methodology, and external validation/preregistration is planned for future studies. Despite these limitations, the study has several strengths. It is among the first to combine fNIRS and machine learning for predicting detailed olfactory subdomain performance in healthy adults, demonstrating feasibility for non-invasive, real-time assessment. The study employed rigorous preprocessing, balanced dataset strategies, and cross-validation to enhance model reliability. Furthermore, feature attribution techniques (SHAP) provided interpretable insights for threshold and discrimination models, linking specific neural response characteristics to functional outcomes. Finally, the use of a culturally adapted YOF test ensures ecological validity for the target population.

### 4.4. Future Directions

Future research should expand and apply this modeling approach to clinical populations, such as individuals with post-viral anosmia, traumatic olfactory loss, or early Alzheimer’s disease to evaluate generalizability [28]. Integrating regression-based models for continuous score prediction and integrating multimodal data (e.g., electroencephalogram, olfactometers) could enhance both predictive performance and physiological interpretability. Longitudinal studies could further assess whether fNIRS-derived biomarkers can monitor disease progression or treatment response [29]. Finally, cross-cultural validation of odor identification tests will be critical to extend applicability beyond the Korean population.

## 5. Conclusions

In healthy, normosmic adults, time-locked fNIRS during YOF tasks contained sufficient information to predict psychophysical olfactory subdomains, providing preliminary, internally validated evidence for a non-invasive, portable assessment approach. Machine learning models trained on prefrontal hemodynamic responses successfully estimated the subcomponents of the YOF test: threshold, discrimination, and identification. Our findings suggest that olfactory-related brain activity measured by fNIRS carries sufficient information to estimate olfactory abilities even within a normosmic population. This work provides preliminary evidence supporting the feasibility of non-invasive, real-time assessment of olfactory function using portable neuroimaging technology. The results open new avenues for future research and potential clinical applications, particularly in early detection and longitudinal monitoring of olfactory dysfunction and neurodegenerative diseases. Further studies involving clinical populations, broader olfactory ranges, and multimodal signal integration are needed to refine and validate this approach.

## Figures and Tables

**Table 1:** Baseline characteristics.

Characteristic	Value
Number of participants enrolled, *n*	100
Number of dropouts, *n*	0
Final number of participants, *n*	100
Mean age, years ± SD	50.90 ± 11.41
Age range, years	21–76
Sex distribution, female *n* (%)	92 (92%)

## Data Availability

The authors confirm that data supporting the findings of this study are available upon reasonable request.

## Supplementary Materials

The following supporting information can be downloaded at https://www.mdpi.com/article/10.3390/diagnostics15192433/s1, Figure S1: Study flow diagram; Table S1: Sex-stratified exploratory results; Checklist S1: TRIPOD-AI Checklist (completed); Checklist S2: STARD 2015 Checklist (completed).

## Abbreviations

AUC	area under the curve
CNN	convolutional neural network
fNIRS	functional near-infrared spectroscopy
LZC	Lempel-Ziv complexity
OD	olfactory dysfunction
SHAP	SHapley Additive exPlanations
UPSIT	University of Pennsylvania Smell Identification Test
YOF	YSK olfactory function
