# Supplementary material for: Diagnostic Efficacy of Olfactory Function Test Using Functional Near-Infrared Spectroscopy with Machine Learning in Healthy Adults: A Prospective Diagnostic-Accuracy (Feasibility/Validation) Study in Healthy Adults with Algorithm Development
Source: Diagnostics (Basel). 2025 Sep 24;15(19):2433. doi: 10.3390/diagnostics15192433 (PMC12523926; doi:10.3390/diagnostics15192433)
Supplement: Supplementary file 1 [file diagnostics-15-02433-s001.zip › diagnostics-3848593-supplementary.pdf]

Supplementary Figure S1. Study flow diagram

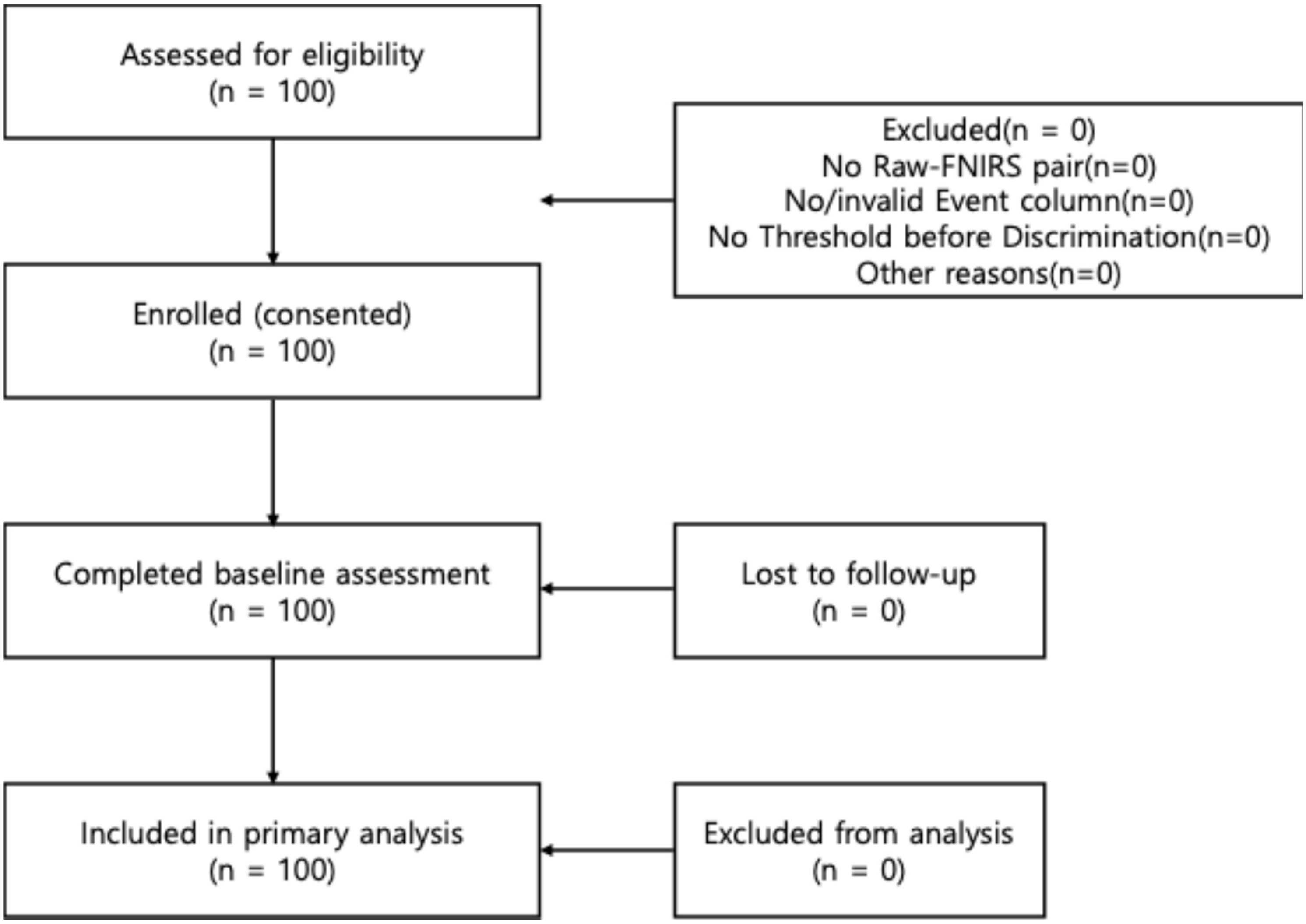

Supplementary Table S1. Sex-stratified exploratory results

	Sex	Overall(n)	ACC	SEN	SPEC	AUC
**Threshold**	Female	92	0.7	0.69	0.71	0.84
	Male	8	1.0	1.0	nan	-
**Discrimination**	Female	92	0.62	0.61	0.62	0.69
	Male	8	1.0	1.0	nan	-
**Identification**	Female	92	0.9	0.875	1.0	1.0
	Male	8	0.91	0.94	0.89	0.96

Version: 11-January-2024

Supplementary Checklist S1: TRIPOD-AI Checklist (completed)

Section/Topic	Item	Development / evaluation[1]	Checklist item	Reportedon page
**TITLE**				
Title	1	D;E	Identify the study as developing or evaluating the performance of a multivariable prediction model, thetarget population, and the outcome to be predicted	p.1, Title
**ABSTRACT**				
Abstract	2	D;E	See TRIPOD+AI for Abstracts checklist	p.1, Abstract
**INTRODUCTION**				
Background	3a	D;E	Explain the healthcare context (including whether diagnostic or prognostic) and rationale for developing or evaluating the prediction model, including references to existing models	p.2, Introduction
	3b	D;E	Describe the target population and the intended purpose of the prediction model in the context of the care pathway, including its intended users (e.g., healthcare professionals, patients, public)	p.2-3, Introduction
	3c	D;E	Describe any known health inequalities between sociodemographic groups	Not Reported
Objectives	4	D;E	Specify the study objectives, including whether the study describes the development or validation of a prediction model (or both)	p.3, Introduction
**METHODS**				
Data	5a	D;E	Describe the sources of data separately for the development and evaluation datasets (e.g., randomisedtrial, cohort, routine care or registry data), the rationale for using these data, and representativeness ofthe data	p.3-4, Methods-Dataset
	5b	D;E	Specify the dates of the collected participant data, including start and end of participant accrual; and, if applicable, end of follow-up	p.3, Methods-Study period
Participants	6a	D;E	Specify key elements of the study setting (e.g., primary care, secondary care, general population) including the number and location of centres	p.3, Methods-Participants, single-centre
	6b	D;E	Describe the eligibility criteria for study participants	p.3, Methods-Preprocessing
	6c	D;E	Give details of any treatments received, and how they were handled during model development orevaluation, if relevant	N/A (olfactory test only, no treatment)
Data preparation	7	D;E	Describe any data pre-processing and quality checking, including whether this was similar across relevant sociodemographic groups	p.4, Methods-Preprocessing
Outcome	8a	D;E	Clearly define the outcome that is being predicted and the time horizon, including how and when assessed, the rationale for choosing this outcome, and whether the method of outcome assessment is consistent across sociodemographic groups	p.4, Methods-Threshold/Discrimination/Identification, TDI <=21
	8b	D;E	If outcome assessment requires subjective interpretation, describe the qualifications and demographiccharacteristics of the outcome assessors	Not Reported
	8c	D;E	Report any actions to blind assessment of the outcome to be predicted	Not Reported
Predictors	9a	D	Describe the choice of initial predictors (e.g., literature, previous models, all available predictors) and any pre-selection of predictors before model building	p.5, Methods-Feature extraction
	9b	D;E	Clearly define all predictors, including how and when they were measured (and any actions to blindassessment of predictors for the outcome and other predictors)	p.5-6, Methods-Feature extraction & feature list
	9c	D;E	If predictor measurement requires subjective interpretation, describe the qualifications and demographiccharacteristics of the predictor assessors	N/A (automatic features, not human-rated
Sample size	10	D;E	Explain how the study size was arrived at (separately for development and evaluation), and justify that the study size was sufficient to answer the research question. Include details of any sample size calculation	Not Reported
Missing data	11	D;E	Describe how missing data were handled. Provide reasons for omitting any data	p.4, Methods-Missing data strategy
Analytical methods	12a	D	Describe how the data were used (e.g., for development and evaluation of model performance) in the analysis, including whether the data were partitioned, considering any sample size requirements	p.6, Methods-CV&hold-out split
	12b	D	Depending on the type of model, describe how predictors were handled in the analyses (functional form, rescaling, transformation, or any standardisation).	p.5, Methods-zscore normalization
	12c	D	Specify the type of model, rationale[2], all model-building steps, including any hyperparameter tuning, and method for internal validation	p.6-7, Methods-Model development, XGboost&RF with tuning

	12d	D;E	Describe if and how any heterogeneity in estimates of model parameter values and model performance was handled and quantified across clusters (e.g., hospitals, countries). See TRIPOD-Cluster for additional considerations[3]	Not reported (single-centre only)
	12e	D;E	Specify all measures and plots used (and their rationale) to evaluate model performance (e.g., discrimination, calibration, clinical utility) and, if relevant, to compare multiple models	p.8-9, Results-ROC, AUC, confusion matrix
	12f	E	Describe any model updating (e.g., recalibration) arising from the model evaluation, either overall or for particular sociodemographic groups or settings	N/A
	12g	E	For model evaluation, describe how the model predictions were calculated (e.g., formula, code, object, application programming interface)	Not Reported
Class imbalance	13	D;E	If class imbalance methods were used, state why and how this was done, and any subsequent methods to recalibrate the model or the model predictions	p.6, Methods-Class weights/SMOTE
Fairness	14	D;E	Describe any approaches that were used to address model fairness and their rationale	Not Reported
Model output	15	D	Specify the output of the prediction model (e.g., probabilities, classification). Provide details and rationale for any classification and how the thresholds were identified	p.6-7, Methods-binary classification, threshold description
Training versus evaluation	16	D;E	Identify any differences between the development and evaluation data in healthcare setting, eligibility criteria, outcome, and predictors	Not Reported
Ethical approval	17	D;E	Name the institutional research board or ethics committee that approved the study and describe the participant-informed consent or the ethics committee waiver of informed consent	p.3, Methods-IRB approval
**OPEN SCIENCE**				
Funding	18a	D;E	Give the source of funding and the role of the funders for the present study	p.14, Acknowledgements
Conflicts of interest	18b	D;E	Declare any conflicts of interest and financial disclosures for all authors	p.14, Declarations
Protocol	18c	D;E	Indicate where the study protocol can be accessed or state that a protocol was not prepared	Not Reported
Registration	18d	D;E	Provide registration information for the study, including register name and registration number, or state that the study was not registered	Not Reported
Data sharing	18e	D;E	Provide details of the availability of the study data	Not Reported
Code sharing	18f	D;E	Provide details of the availability of the analytical code[4]	Not Reported
**PATIENT & PUBLIC INVOLVEMENT**				
Patient & Public Involvement	19	D;E	Provide details of any patient and public involvement during the design, conduct, reporting,interpretation, or dissemination of the study or state no involvement.	N/A
**RESULTS**				
Participants	20a	D;E	Describe the flow of participants through the study, including the number of participants with and without the outcome and, if applicable, a summary of the follow-up time. A diagram may be helpful.	Fig.1, Results
	20b	D;E	Report the characteristics overall and, where applicable, for each data source or setting, including thekey dates, key predictors (including demographics), treatments received, sample size, number of outcome events, follow-up time, and amount of missing data. A table may be helpful. Report any differences across key demographic groups.	Table1, Results
	20c	E	For model evaluation, show a comparison with the development data of the distribution of importantpredictors (demographics, predictors, and outcome).	Not reported
Model development	21	D;E	Specify the number of participants and outcome events in each analysis (e.g., for model development, hyperparameter tuning, model evaluation)	p.6-7, Methods-CV splits, also Table1
Model specification	22	D	Provide details of the full prediction model (e.g., formula, code, object, application programminginterface) to allow predictions in new individuals and to enable third-party evaluation and implementation, including any restrictions to access or re-use (e.g., freely available, proprietary)[5]	Not reported
Model performance	23a	D;E	Report model performance estimates with confidence intervals, including for any key subgroups (e.g.,sociodemographic). Consider plots to aid presentation.	p.9, Results-ROC curve, AUC with CI
	23b	D;E	If examined, report results of any heterogeneity in model performance across clusters. See TRIPOD Cluster for additional details[3].	Not reported
Model updating	24	E	Report the results from any model updating, including the updated model and subsequent performance	N/A
**DISCUSSION**				
Interpretation	25	D;E	Give an overall interpretation of the main results, including issues of fairness in the context of the objectives and previous studies	p.11-12, Discussion
Limitations	26	D;E	Discuss any limitations of the study (such as a non-representative sample, sample size, overfitting,missing data) and their effects on any biases, statistical uncertainty, and generalizability	p.12, Discussion-Limitations

Usability of the model in the context of current care	27a	D	Describe how poor quality or unavailable input data (e.g., predictor values) should be assessed and handled when implementing the prediction model	Not reported
	27b	D	Specify whether users will be required to interact in the handling of the input data or use of the model,<br>and what level of expertise is required of users	Not reported
	27c	D;E	Discuss any next steps for future research, with a specific view to applicability and generalizability of<br>the model	p.13, Discussion

Supplementary Checklist S2: STARD 2015 Checklist (completed)

Section & Topic	No.	Item	(N/A)
**TITLE OR ABSTRACT**			
	1	Identification as a study of diagnostic accuracy using at least one measure of accuracy (such as sensitivity, specificity, predictive values, or AUC)	Reported
**ABSTRACT**			
	2	Structured summary of study design, methods, results, and conclusions (for specific guidance, see STARD for Abstracts)	Reported
**INTRODUCTION**			
	3	Scientific & clinical background, including the intended use and clinical role of the index test	Reported
	4	Study objectives and hypotheses	Reported
**METHODS**			
Study design	5	Whether data collection was planned before the index test and reference standard were performed (prospective study) or after (retrospective study)	Reported
Participants	6	Eligibility criteria	Reported
	7	On what basis potentially eligible participants were identified (such as symptoms, results from previous tests, inclusion in registry)	Reported
	8	Where and when potentially eligible participants were identified (setting, location and dates)	Reported
	9	Whether participants formed a consecutive, random or convenience series	Not Reported
Test methods	10a	Index test, in sufficient detail to allow replication	Reported
	10b	Reference standard, in sufficient detail to allow replication	Reported
	11	Rationale for choosing the reference standard (if alternatives exist)	Reported
	12a	Definition of and rationale for test positivity cut-offs or result categories of the index test, distinguishing pre-specified from exploratory	Reported
	12b	Definition of and rationale for test positivity cut-offs or result categories of the reference standard, distinguishing pre-specified from exploratory	Reported
	13a	Whether clinical information and reference standard results were available to the performers/readers of the index test	Not Reported
	13b	Whether clinical information and index test results were available to the assessors of the reference standard	Not Reported
Analysis	14	Methods for estimating or comparing measures of diagnostic accuracy	Reported
	15	How indeterminate index test or reference standard results were handled	Not Reported
	16	How missing data on the index test and reference standard were handled	Reported
	17	Any analyses of variability in diagnostic accuracy, distinguishing pre-specified from exploratory	Not Reported
	18	Intended sample size and how it was determined	Not Reported

**RESULTS**			
Participants	19	Flow of participants, using a diagram. Include the figure number (preferably figure 1) or page number	Reported
	20	Baseline demographic and clinical characteristics of participants	Reported
	21a	Distribution of severity of disease in those with the target condition	Not Reported
	21b	Distribution of alternative diagnoses in those without the target condition	Not Reported
	22	Time interval and any clinical interventions between index test and reference standard	N/A
Test results	23	Cross tabulation of the index test results (or their distribution) by the results of the reference standard	Reported
	24	Estimates of diagnostic accuracy and their precision (such as 95% confidence intervals)	Reported
	25	Any adverse events from performing the index test or the reference standard	N/A
**DISCUSSION**			
	26	Study limitations, including sources of potential bias, statistical uncertainty, and generalisability	Reported
	27	Implications for practice, including the intended use and clinical role of the index test	Reported
**OTHER INFORMATION**			
	28	Registration number and name of registry	Not Reported
	29	Where the full study protocol can be accessed	Not Reported
	30	Sources of funding and other support; role of funders	Reported

*N/A stands for not applicable and may be a reasonable choice depending on the type of study performed
